# VEGF Regulation of Angiogenic Factors via Inflammatory Signaling in Myeloproliferative Neoplasms

**DOI:** 10.3390/ijms22136671

**Published:** 2021-06-22

**Authors:** Tijana Subotički, Olivera Mitrović Ajtić, Emilija Živković, Miloš Diklić, Dragoslava Đikić, Milica Tošić, Bojana Beleslin-Čokić, Teodora Dragojević, Mirjana Gotić, Juan F. Santibanez, Vladan Čokić

**Affiliations:** 1 Department of Molecular Oncology, Institute for Medical Research, University of Belgrade, 11000 Belgrade, Serbia; oliveram@imi.bg.ac.rs (O.M.A.); ema.zivkovic55@gmail.com (E.Ž.); milos.diklic@imi.bg.ac.rs (M.D.); dragoslava@imi.bg.ac.rs (D.Đ.); milica.tosic@imi.bg.ac.rs (M.T.); teodora.dragojevic@imi.bg.ac.rs (T.D.); jfsantibanez@imi.bg.ac.rs (J.F.S.); vl@imi.bg.ac.rs (V.Č.); 2 Clinic for Endocrinology, Diabetes and Metabolic Diseases, Genetic Laboratory, Clinical Center of Serbia, 11000 Belgrade, Serbia; bojanabbc06@yahoo.com; 3 Clinic of Hematology, Clinical Center of Serbia, 11000 Belgrade, Serbia; miragotic@yahoo.com; 4 School of Medicine, University of Belgrade, 11000 Belgrade, Serbia; 5 Centro Integrativo de Biología y Química Aplicada, Universidad Bernardo O’Higgins, Santiago 8370993, Chile

**Keywords:** VEGF, IL-6, myeloproliferative neoplasm, Ruxolitinib, angiogenesis

## Abstract

Background: Chronic inflammation has been recognized in neoplastic disorders, including myeloproliferative neoplasm (MPN), as an important regulator of angiogenesis. Aims: We investigated the influence of vascular endothelial growth factor (VEGF) and pro-inflammatory interleukin-6 (IL-6) on the expression of angiogenic factors, as well as inflammation-related signaling in mononuclear cells (MNC) of patients with MPN and *JAK2*V617F positive human erythroleukemic (HEL) cells. Results: We found that IL-6 did not change the expression of angiogenic factors in the MNC of patients with MPN and HEL cells. However, IL-6 and the JAK1/2 inhibitor Ruxolitinib significantly increased angiogenic factors—endothelial nitric oxide synthase (eNOS), VEGF, and hypoxia-inducible factor-1 alpha (HIF-1α)—in patients with polycythemia vera (PV). Furthermore, VEGF significantly increased the expression of HIF-1α and eNOS genes, the latter inversely regulated by PI3K and mTOR signaling in the MNC of primary myelofibrosis (PMF). VEGF and inhibitors of inflammatory JAK1/2, PI3K, and mTOR signaling reduced the eNOS protein expression in HEL cells. VEGF also decreased the expression of eNOS and HIF-1α proteins in the MNC of PMF. In contrast, VEGF increased eNOS and HIF-1α protein expression in the MNC of patients with PV, which was mediated by the inflammatory signaling. VEGF increased the level of IL-6 immunopositive MNC of MPN. In summary, VEGF conversely regulated gene and protein expression of angiogenic factors in the MNC of PMF, while VEGF increased angiogenic factor expression in PV mediated by the inflammation-related signaling. Conclusion: The angiogenic VEGF induction of IL-6 supports chronic inflammation that, through positive feedback, further promotes angiogenesis with concomitant JAK1/2 inhibition.

## 1. Introduction

Myeloproliferative neoplasms (MPNs) are characterized by clonal proliferation of mature blood elements from several myeloid lineages associated with increased angiogenesis [1]. Angiogenesis, that is increased in MPNs, is measured by the expression of vascular endothelial growth factor (VEGF) and hypoxia-inducible factor-1 alpha (HIF-1α) [2,3]. We demonstrated that microvessel density was increased and in positive correlation with angiogenic factors VEGF, basic fibroblast growth factor, and bone marrow fibrosis in MPNs [4]. Moreover, we revealed that the expression of HIF-1α, VEGF, and endothelial nitric oxide synthase (eNOS) proteins were generally increased in granulocytes and immunopositive CD34+ cells of MPN, with no steady changed levels in bone marrow [5]. Participation of inflammatory cells is supported by increased inteleukin-6 (IL-6) cytokine in plasma and bone marrow stroma of MPNs, which is dependent on JAK2V617F [6]. These observations support further studies of inflammation-dependent angiogenesis with emphasis on proliferation-related signaling pathways as a hallmark of MPN.

Excessive myeloproliferation in MPN is characterized by constitutive activation of JAK2/STAT3 signaling in MPN, while combined inhibition of PI3K/mammalian target of rapamycin (mTOR) and JAK2 signaling pathways reduces the extent of disease and prolonged survival [7]. Furthermore, angiogenesis is also regulated by modulation of the mTOR signaling pathway linked to HIF-1α and VEGF [6]. When we analyzed mTOR signaling pathway-related genes, PI3K/AKT regulators were preferentially upregulated in circulatory CD34+ cells of MPN [8]. The observation of linked PI3K/AKT/mTOR pathway requests the cells with constitutive activation of JAK2/STAT3 signaling to determine the angiogenic factors response.

One of the factors that induce angiogenic VEGF expression via JAK2/STAT3 signaling is pro-inflammatory IL-6 [9]. IL-6 also induces the dose-dependent release of VEGF from platelets, further linking the inflammatory process to angiogenesis [10]. We already reported elevated plasma levels of IL-6 in patients with MPN and that IL-6 stimulated JAK2/STAT3 and AKT signaling in polycythemia vera (PV) and primary myelofibrosis (PMF) [6]. IL-6 is produced predominantly by monocytes, macrophages, and T cells [11]. These reports support chronic inflammation as a promoter of angiogenesis.

In this study, we expanded our previous research by examining the mechanism of IL-6 induction of angiogenic factors via proliferation-related JAK1/2, PI3K, and mTOR signaling in JAK2V617F positive HEL cells and mononuclear cells (MNC contains lymphocytes (T cells) and monocytes) of patients with MPN. Moreover, we studied the JAK1/2, PI3K, and mTOR mediated induction of HIF-1α and eNOS by VEGF on gene and protein levels in MNC parallel with the observation of AKT and mTOR phosphorylation. We also examined feedback of IL-6 induction by VEGF in MNC and HEL cells. By this approach, we wanted to reveal the interaction between inflammation and angiogenesis through proliferation related signaling pathways in MPN.

## 2. Results

### 2.1. IL-6 Effect on Angiogenic Factors in Mononuclear Cells of MPN

**Figure 1:**
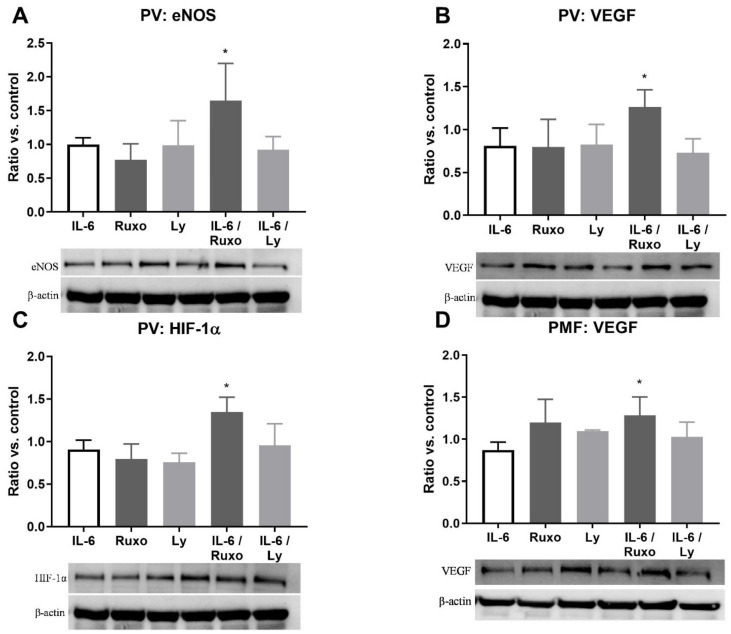
Interleukin-6 (IL-6) induction of angiogenic factors in mononuclear cells (MNC) of myeloproliferative neoplasms (MPN). Densitometry revealed protein expression determined by Western blotting and presented as a ratio of treated to non-treated MNC (Control). The MNC were treated for 1 h by 20 ng/mL IL-6 with or without 1 µM JAK1/2 inhibitor Ruxolitinib and 5 µM PI3K inhibitor LY294002 in (**A**–**C**) polycythemia vera (PV) and (**D**) primary myelofibrosis (PMF). Values are mean ± SEM (*n* = 3). * *p* < 0.05 vs. IL-6.

Pro-inflammatory IL-6 does not cause a significant increase in the expression of angiogenic factors in MNC of patients with MPN (Figure 1). Moreover, IL-6 and JAK1/2 inhibitor Ruxolitinib significantly increased angiogenic factors—eNOS (Figure 1A), VEGF (Figure 1B), and HIF-1α (Figure 1C—in PV patients. In addition, there is an increased expression of VEGF after treatment with IL-6 and Ruxolitinib in PMF patients (Figure 1D). The mutual effects of Ruxolitinib and IL-6 have not been observed in MNC of essential thrombocythemia (ET) (not shown). However, IL-6 does not change the level of angiogenic factors in the MNC of MPN.

### 2.2. IL-6 Effect on Angiogenic Factors and Inflammation-Related Signaling Pathways in JAK2V617F Positive HEL Cells

**Figure 2:**
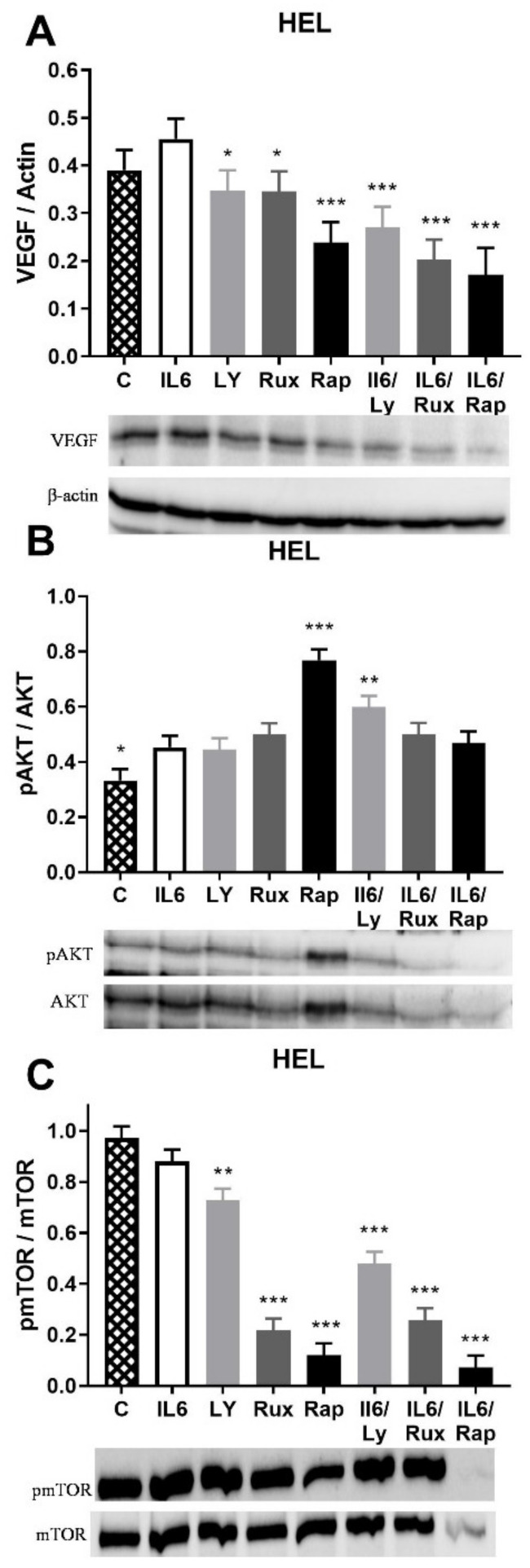
Interleukin-6 (IL-6) induction of angiogenic factors and related signalling pathways in JAK2V617F positive human erythroleukemic (HEL) cells. Densitometry revealed protein expression determined by Western blotting and presented as a ratio to Actin of total protein levels. The HEL cells were treated for 1 h by 20 ng/mL IL-6 with or without 1 µM JAK1/2 inhibitor Ruxolitinib, 5 µM PI3K inhibitor LY294002 and 100 ng/mL mTOR inhibitor Rapamycin and levels of (**A**) VEGF, (**B**) phospho AKT, and (**C**) phospho mTOR were determined. Values are mean ± SEM (*n* = 3). * *p* < 0.05, ** *p* < 0.01, *** *p* < 0.001 vs. IL-6. * *p* < 0.05, ** *p* < 0.01, *** *p* < 0.001 vs. VEGF vs. Control.

mTOR inhibition led to a significant reduction in VEGF expression in HEL cells with JAK2V617F (Figure 2A). JAK1/2, PI3K, and mTOR inhibition reduced the IL-6 stimulated VEGF expression (Figure 2A). The IL-6 failed to influence eNOS and HIF-1α protein expression in HEL cells (not shown). The AKT signaling pathway was activated by IL-6 as well as by inhibition of JAK1/2, PI3K, and mTOR signaling, though the latter had the most prominent effect (Figure 2B). Moreover, the PI3K inhibitor Ly294002 enhanced IL-6 stimulation of AKT signaling (Figure 2B) as well as STAT5 phosphorylation (*p* < 0.001, not shown). JAK1/2, PI3K, and mTOR inhibitors significantly dephosphorylated mTOR signaling either individually or in combination with IL-6 (Figure 2C). IL-6 did not change the angiogenic factors level and phosphorylation of STAT5 and mTOR in HEL cells, while slightly stimulating AKT signaling (not shown). The JAK1/2, PI3K, and mTOR inhibition generally did not affect the level of the angiogenic factor, while diversely regulating AKT and mTOR signaling in HEL cells.

### 2.3. VEGF Induction of Angiogenic Factors Gene Expression in MNC of MPN

**Figure 3:**
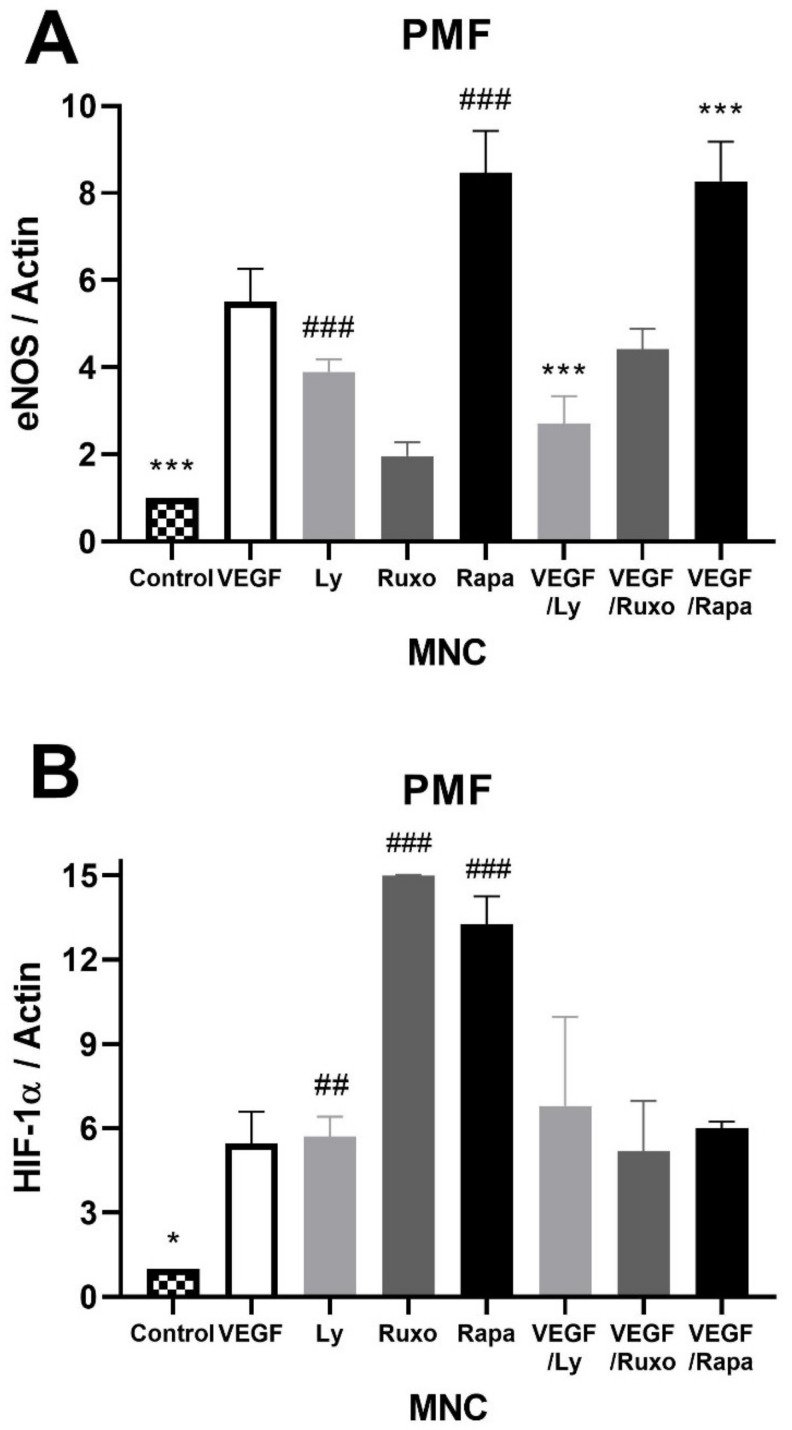
Vascular endothelial growth factor (VEGF) induction of angiogenic factors gene expression in primary myelofibrosis (PMF). Real time qPCR determination of endothelial nitric oxide synthase (eNOS) and hypoxia inducible factor 1-α (HIF-1α) presented as a ratio to Actin. The mononuclear cells (MNC) were treated for 1 h with 10 µg/mL VEGF with or without 1 µM JAK1/2 inhibitor Ruxolitinib, 5 µM PI3K inhibitor LY294002 and 100 ng/mL mTOR inhibitor Rapamycin and levels of (**A**) eNOS, and (**B**) HIF-1α were determined. Values are mean ± SEM (*n* = 3). * *p* < 0.05, *** *p* < 0.001 vs. VEGF treated cells. ^##^ *p* < 0.01, ^###^ *p* < 0.001 vs. Control.

After mutual treatment with VEGF and inhibitors of inflammation-related signaling pathways, the gene expression of the angiogenic factors HIF-1α and eNOS was monitored in HEL cells and MNC of MPN patients. VEGF significantly increased the gene expression of angiogenic factors HIF-1α (*p* < 0.05) and eNOS (*p* < 0.001) in the MNC of patients with PMF (Figure 3). In addition, PI3K and mTOR inhibitors also increased eNOS gene expression, while JAK1/2, PI3K, and mTOR inhibitors increased HIF-1α gene expression (Figure 3A,B). In addition, the PI3K inhibitor prevented VEGF simulation of eNOS gene expression while the mTOR inhibitor augmented VEGF stimulation of eNOS gene expression (Figure 3A). VEGF transcriptional activation of the angiogenic factors was inversely controlled by PI3K and mTOR signaling for the eNOS gene only.

### 2.4. VEGF Induction of Angiogenic Factors and Inflammation-Related Signaling Pathways in HEL Cells

**Figure 4:**
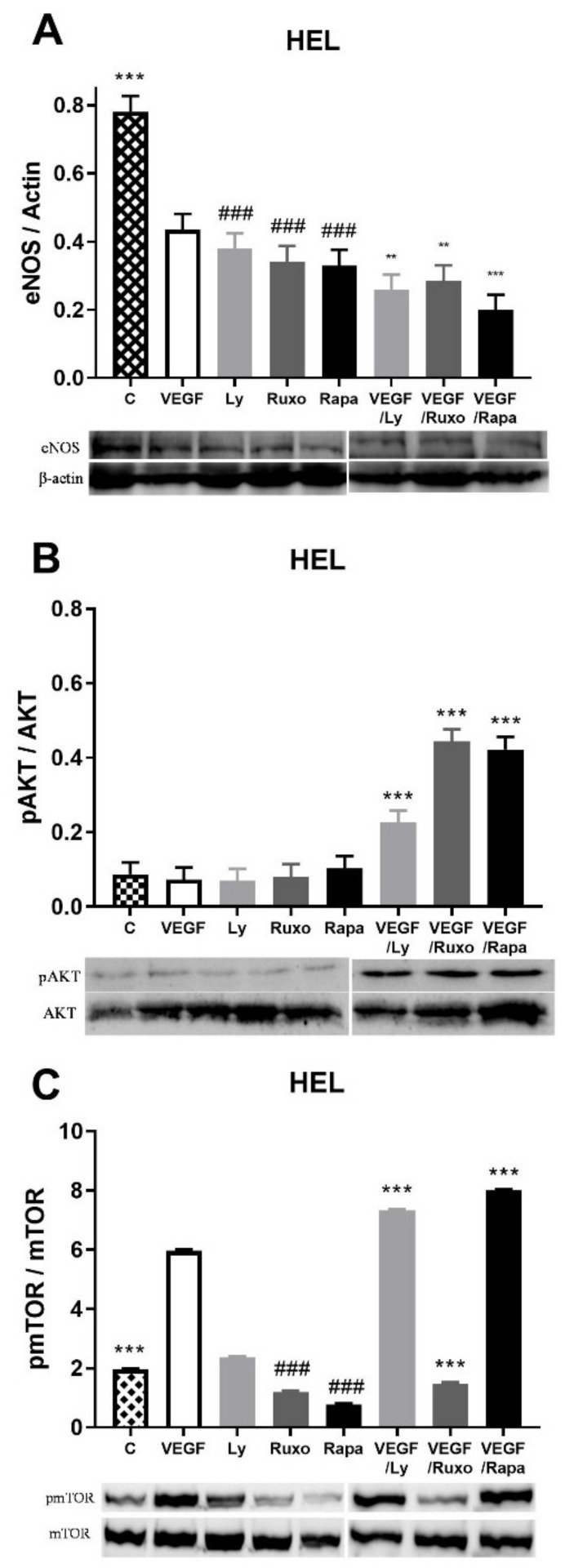
Vascular endothelial growth factor (VEGF) induction of angiogenic factors and related signalling pathways in *JAK2*V617F positive human erythroleukemic cells (HEL). Densitometry revealed protein expression determined by Western blotting and presented as a ratio to Actin of total protein levels. The HEL cells were treated for 1 h with 10 µg/mL VEGF with or without 1 µM JAK1/2 inhibitor Ruxolitinib, 5 µM PI3K inhibitor LY294002 and 100 ng/mL mTOR inhibitor Rapamycin and levels of (**A**) endothelial nitric oxide synthase (eNOS), (**B**) phospho AKT, and (**C**) phospho mTOR were determined. Values are mean ± SEM (*n* = 3). ** *p* < 0.01, *** *p* < 0.001 vs. VEGF; ^###^ *p* < 0.001 vs. Control.

We wanted to examine the influence of VEGF on the expression of the other two angiogenic factors as well as the inflammation-related signaling pathways in HEL cells with JAK2V617F. VEGF decreased eNOS protein expression as well as JAK1/2, PI3K, and mTOR inhibitors either individually or mutually with VEGF (Figure 4A). In the case of HIF-1α protein expression, neither VEGF nor inhibitors led to a significant change in its expression (not shown). In the case of inflammation-related signaling pathways, significant activation of the AKT signaling pathway occurred during the combined action of VEGF and JAK1/2, PI3K, and mTOR inhibitors (Figure 4B). In addition, STAT5 signaling is uniform and independent of the presence of VEGF or the inhibitors of inflammation-related signaling pathways (not shown). VEGF largely increased the activation of mTOR signaling, while JAK1/2 and mTOR inhibitors reduced it (Figure 4C). Further, PI3K and mTOR inhibitors supported VEGF activation of mTOR signaling, but Ruxolitinib prevented it in HEL cells (Figure 4C). JAK1/2, PI3K, and mTOR inhibitors generally decreased angiogenic factors in HEL cells, while VEGF decreased the eNOS and stimulated mTOR signaling.

### 2.5. VEGF Induction of Angiogenic Factors and Inflammation-Related Signaling Pathways in MPN

**Figure 5:**
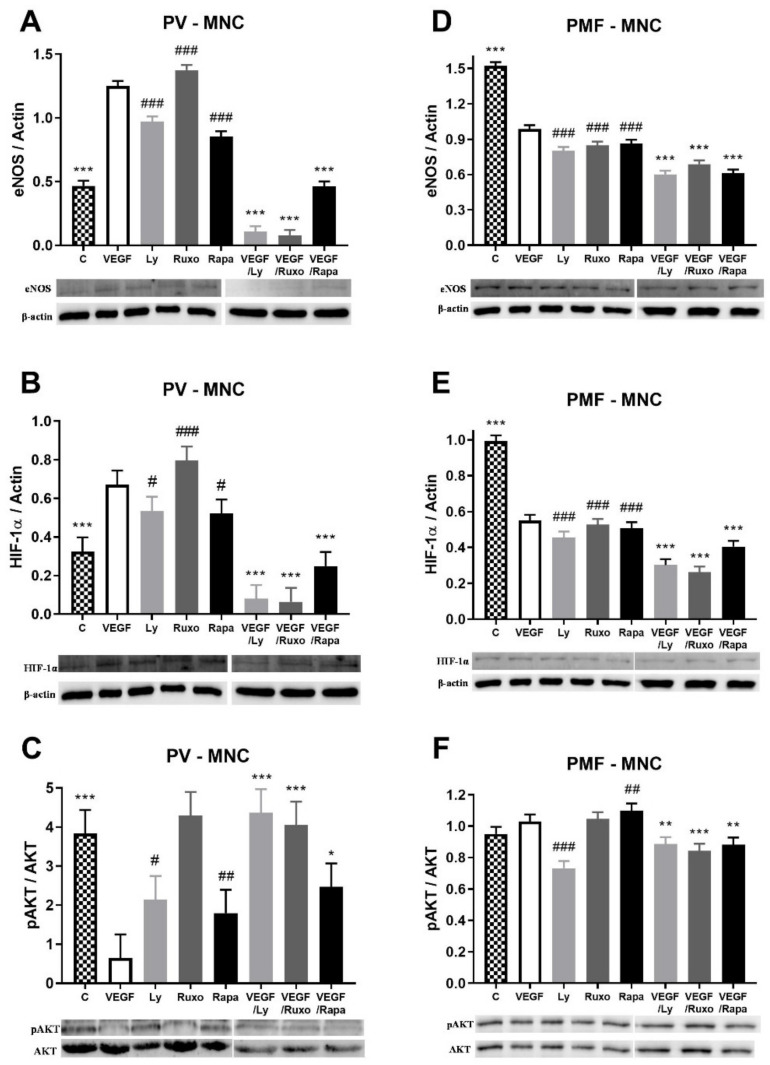
Vascular endothelial growth factor (VEGF) induction of angiogenic factors and related signalling pathways in myeloproliferative neoplasms (MPN). Densitometry revealed protein expression determined by Western blotting and presented as a ratio to Actin of total protein levels. The MNC were treated for 1 h with 10 µg/mL VEGF with or without 1 µM JAK1/2 inhibitor Ruxolitinib, 5 µM PI3K inhibitor LY294002 and 100 ng/mL mTOR inhibitor Rapamycin. The levels of (**A**) endothelial nitric oxide synthase (eNOS), (**B**) phospho AKT, and (**C**) hypoxia inducible factor 1-α (HIF-1α) in polycythemia vera (PV) were determined, while (**D**) eNOS, (**E**) phospho AKT, and (**F**) HIF-1α were determined in primary myelofibrosis (PMF). Values are mean ± SEM (*n* = 3). * *p* < 0.05, ** *p* < 0.01, *** *p* < 0.001 vs. VEGF; ^#^ *p* < 0.05, ^##^ *p* < 0.01, ^###^ *p* < 0.001 vs. Control.

The influence of VEGF on angiogenic factors and inflammation-related signaling pathways was also monitored in the MNC of MPN patients. VEGF increased the expression of angiogenic eNOS and HIF-1α proteins in the MNC of PV patients similarly to JAK1/2, PI3K, and mTOR inhibitors (Figure 5A,B). Conversely, VEGF in combination with the inhibitors led to a significant decrease in eNOS and HIF-1α expression in PV (Figure 5A,B). VEGF largely dephosphorylated AKT signaling in MNC of PV patients similar to PI3K and mTOR inhibitors (Figure 5C). The JAK1/2, PI3K, and mTOR inhibitors individually diminished the VEGF-mediated deactivation of AKT signaling (Figure 5C). In contrast, VEGF decreased the expression of eNOS and HIF-1α proteins in the MNC of PMF patients similarly to JAK1/2, PI3K, and mTOR inhibitors (Figure 5D,E). This VEGF-induced reduction of the angiogenic factors was further aggravated by the JAK1/2, PI3K, and mTOR inhibitors (Figure 5D,E). Activation of AKT signaling was inversely regulated by PI3K and mTOR inhibitors, but all inhibitors reduced the VEGF effect on AKT signaling (Figure 5F). No significant difference in the expression of both angiogenesis factors and the observed signaling pathways was observed in the MNC of ET patients during VEGF treatment (not shown). Additionally, there was no significant change in STAT5 and mTOR signaling during VEGF treatment in MPN patients (not shown). VEGF increased the angiogenic factors in the MNC of PV but reduced them in PMF, followed and supported by the inhibition of JAK1/2, PI3K, and mTOR signaling.

### 2.6. VEGF Induction of IL-6 in MPN

**Figure 6:**
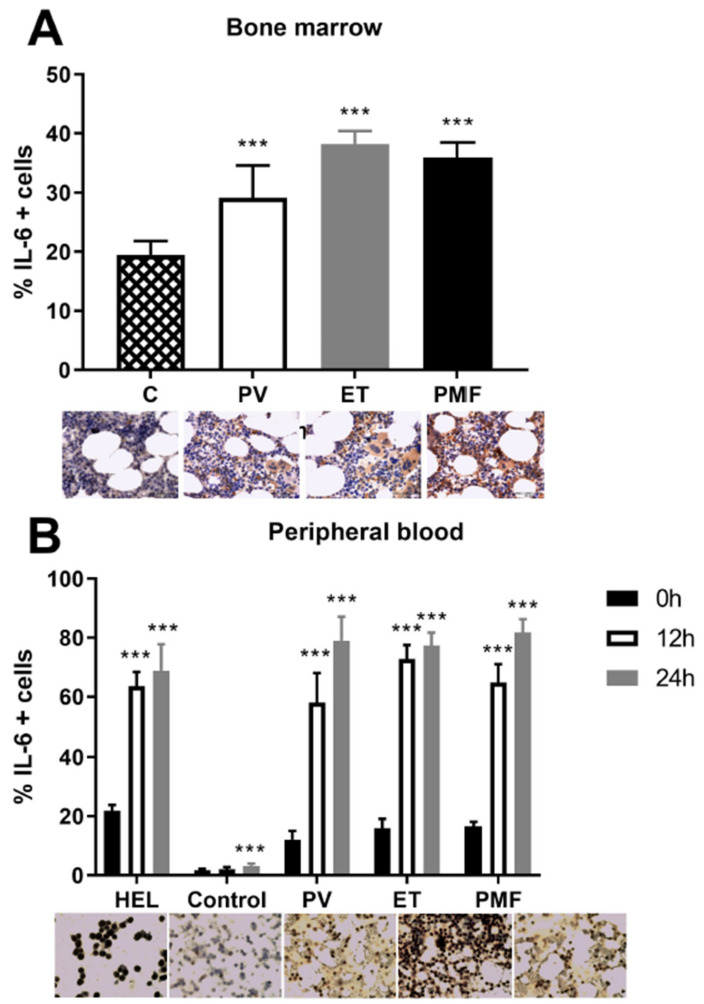
Percentage of IL-6 positive cells in (**A**) bone marrow of polycythemia vera (PV), essential thrombocythemia (ET), and primary myelofibrosis (PMF) determined by immunohistochemistry (IHC, *** *p* < 0.001 vs. control) and (**B**) human erythroleukemic (HEL with JAK2V617F) cells, peripheral blood derived MNC of healthy controls, PV, ET, and PMF treated for 12 and 24 h with 10 µg/mL VEGF determined by immunocytochemistry (ICC). ICC slides correspond to columns in graph, after 24 h of treatment by VEGF. *** *p* < 0.001 vs. non-treated cells. Values are mean ± SEM (*n* = 3).

Since we have previously shown that the levels of pro-inflammatory cytokine IL-6 are elevated in the plasma and bone marrow of MPN patients [12], in accordance with the JAK2V617F mutant allele burden, we also wanted to examine the total degree of its expression in the bone marrow of MPN patients. Immunohistochemical analysis of IL-6 positive cells confirmed that a percentage of this pro-inflammatory cytokine was significantly elevated in the bone marrow of MPN patients compared to controls (Figure 6A). We also monitored the IL-6 positive MNC of MPN patients after treatment with VEGF for 12 and 24 h. Regardless of the duration of VEGF treatment, all MPN patients had increased IL-6 positive cells relative to untreated MNC and expression was higher compared to healthy controls (Figure 6B). The same applies to HEL cells (Figure 6B). VEGF increased the level of IL-6 positive MNC in peripheral blood of MPN.

## 3. Discussion

According to the presented results, IL-6 does not change the level of angiogenic factors in the MNC of MPN and HEL cells, as well as the phosphorylation of STAT5 and mTOR in HEL cells, while it slightly stimulated AKT signaling. Inhibition of inflammatory JAK1/2, PI3K, and mTOR signaling decreased eNOS protein levels, while VEGF decreased eNOS protein levels and stimulated mTOR signaling. The transcriptional activation of the eNOS gene by VEGF was prevented by PI3K and enhanced by mTOR signaling. Furthermore, VEGF increased the expression of eNOS and HIF-1α proteins in the MNC of PV but reduced it in PMF. JAK1/2, PI3K, and mTOR inhibitors prevented the VEGF-stimulated expression of eNOS and HIF-1α proteins in PV, while exaggerating VEGF inhibition of eNOS and HIF-1α protein expression in PMF. VEGF increased the level of IL-6 positive HEL cells and MNC in the peripheral blood of MPN.

Evaluations of aberrant cytokine expression revealed that patients with PMF, PV, and ET had significantly elevated levels of IL-6, IL-8, and VEGF [12,13,14]. Moreover, IL-6 induces JAK/STAT pathway activation, IL-8 affects the tumor microenvironment, and VEGF promotes angiogenesis [15]. We already demonstrated that bone marrow fibrosis in patients with MPN was significantly associated with IL-8 [4]. We expanded the study to the interaction between inflammatory IL-6 and angiogenic VEGF in the MNC of MPN. The role of VEGF in hematopoiesis and angiogenesis has been already confirmed in hematologic malignancies [16]. Elevated levels of VEGF were already demonstrated in the serum and bone marrow of patients with MPN [17,18,19]. A secreted VEGF is thought to contribute to the MPN progression by autocrine or paracrine mechanisms [1]. The expression of VEGF and its receptors increases in the bone marrow of patients with MPN, especially PMF, which could be inversely correlated with survival [20]. These reports confirmed the stimulation of inflammatory and angiogenic factors in MPN.

IL-6 is a proinflammatory cytokine involved in the stimulation of angiogenesis of the tumor microenvironment, as well as in the enhancement of endothelial cell proliferation and migration [21,22]. We previously showed elevated plasma IL-6 levels in MPN patients depending on the presence of the JAK2V617F mutation in patients with ET and PMF [6]. We also found that inhibition of JAK1/2 prevented IL-6 activation of STAT3 and AKT pathways in PV granulocytes and HEL cells. Furthermore, we have shown that JAK1/2 inhibitors also block the IL-6 activation of the AKT pathway in PMF granulocytes [23]. We have now presented that Ruxolitinib significantly increased angiogenic factors—HIF-1α, eNOS, and VEGF in the presence of IL-6—while it only significantly increased the VEGF in the MNC of PMF patients. Therefore, the JAK1/2 inhibitor Ruxolitinib can stimulate angiogenic factors during chronic inflammation that supports the progression of fibrosis in MPN.

Previously, it was shown that IL-6 stimulates MAPK, PI3K-AKT, and STAT3 phosphorylation, while the latter was not prevented by the inhibition of MAPK and PI3K signaling [24]. Additionally, IL-6 induced MAPK phosphorylation was partially blocked by inhibition of PI3K signaling, whereas PI3K-AKT phosphorylation was not prevented by the inhibition of MAPK signaling [25]. We observed that IL-6 slightly activated the AKT signaling pathway as well as JAK1/2, PI3K, and mTOR inhibitors in HEL cells. The PI3K inhibitor Ly294002 enhanced IL-6 stimulation of AKT signaling. Furthermore, inhibitors of all three signaling pathways dephosphorylated the mTOR signaling pathway regardless of the presence of IL-6. On the other hand, Kleppe et al. identified the inflammatory cytokine IL-6 produced by granulocytes of patients with PMF with constitutive STAT3 activation [26]. Inflammatory and proliferation-related signaling pathways, with linked activities, were stimulated and can be an additional therapeutical target besides constitutively activated JAK2-STAT3 signaling.

IL-6 levels are increased in PMF, with a positive correlation between IL-6 and angiogenesis in the bone marrow of patients with MPN [12]. We confirmed an increased expression of IL-6 in the bone marrow of examined patients with MPN [6], while VEGF increased the level of IL-6 positive MNC. It has previously been shown that there is a correlation of VEGF expression with IL-6 and its receptors in tumor cells, which is associated with poor survival of individuals with HER2-invasive ductal carcinoma [27]. This is related to the fact that IL-6 and its receptors are associated with an increased metastatic capacity [28] and the promotion of angiogenesis [29]. In addition, the potential of IL-6 in initiating VEGF expression has been shown in several cancer cells [30,31]. Thus, the anti-IL-6 antibody siltuximab has been shown to reduce STAT3 activation and angiogenesis in IL-6-producing xenografts of intraperitoneal ovarian cancer and reduces VEGF levels in patients with ovarian cancer [32]. The evaluation of IL-6 levels in patients with MPN before and during conventional and new therapies may support future clinical trials that will be able to manage the control of angiogenic factors during disease progression.

## 4. Materials and Methods

### 4.1. HEL 92.1.7 Cell Line

The HEL 92.1.7 cells with a homozygous expression of *JAK2*V617F were cultivated in an RPMI- 1640 medium (Biowest, Nuaillé, France) containing 10% fetal bovine serum (FBS, Biowest) and 1% penicillin-streptomycin (Biowest) at 37 °C in a 5% CO_2_ humidified atmosphere. Next, the HEL 92.1.7 cells were preincubated for 1 h with 1 μM ruxolitinib (RUXO, JAK1/2 inhibitor, Cayman Chemical Company, Ann Arbor, MI, USA), 5 μM Ly294002 (PI3K inhibitor, Cell Signalling Technology, Inc., Danvers, Massachusetts), or 100 ng/mL Rapamycin (RAPA, mammalian target of rapamycin (mTOR) inhibitor, Calbiochem, EMD Millipore Corp., Billerica, MA, USA) and treated for 1 h with IL6 (20 ng/mL, Miltenyi Biotec, Bergisch Gladbach, Germany) or VEGF (10 µg/mL, Elabscience, Wuhan, China). After treatment, the HEL cells were washed once in PBS, incubated in RIPA lysis buffer at 4 °C for 45 min, and centrifuged on 10,000× *g* at 4 °C for 15 min.

### 4.2. Patients

Peripheral blood was obtained from 3 healthy controls and 24 patients diagnosed with MPN according to the World Health Organization (WHO) classification. All of the donors signed the consent form approved by a local ethical committee in accordance with the Declaration of Helsinki. The samples were collected in disodium EDTA and granulocytes were separated using a lymphocyte separation medium (LSM, Capricorn Scientific GmbH, Ebsdorfergrund, Germany) and lysing solution (0.15 M NH_4_Cl, 0.1 mM Na_2_EDTA, 12 mM NaHCO_3_). For immunoblotting and PCR analyses, we had 3 healthy controls and 24 MPN patients: 9 for ET, 7 for PV, and 8 for PMF. The isolated MNC were washed twice in phosphate-buffered saline (PBS) and resuspended in the RPMI-1640 medium (Biowest, Nuaillé, France), preincubated for 1 h with 1 μM ruxolitinib (RUXO), 5 μM Ly294002, or 100 ng/mL Rapamycin (RAPA) and treated for 1 h with IL-6 (20 ng/mL) or VEGF (10 µg/mL). After treatment, the MNC were washed once in PBS, incubated in RIPA lysis buffer at 4 °C for 45 min, and centrifuged on 10,000× *g* at 4 °C for 15 min, or genomic DNA extraction was performed as previously reported [9].

### 4.3. Western Blotting

Proteins from MPN-derived MNC were isolated and processed as previously reported [9]. Equal amounts of protein (30 μg) were run on polyacrylamide gels and transferred to polyvinylidene difluoride membranes. The membranes were blocked with 4% milk (Serva Electrophoresis GmbH, Heidelberg, Germany) for 1 h at room temperature and probed with primary antibodies directed against HIF-1α (Elabscience, Wuhan, China), VEGF (Elabscience), eNOS (Elabscience), β-actin (R&D Systems, Inc, Minneapolis, Minnesota), phospho-STAT5 (R&D Systems), STAT5 (R&D Systems), phospho-AKT (R&D Systems), AKT (R&D Systems), pmTOR (Cell Signaling Technology Inc., Beverly, USA) and mTOR (Cell Signalling Technology). Peroxidase-conjugated goat anti-rabbit immunoglobulin (R&D Systems) was used as a secondary antibody, except goat anti-mouse immunoglobulin (R&D Systems) was used for β-actin. The protein levels were imaged with a ChemiDoc Imaging System (Bio-Rad Laboratories, Hercules, CA, USA) and estimated by densitometric scanning of the blots using the Image Lab (Bio-Rad Laboratories, Inc. Version 6.0.0.25) software tool and normalized to β-actin.

### 4.4. Real-Time Quantitative PCR

Quantitative real-time PCR analyses of human HIF-1α gene was performed using forward 5′ GGC AGG AAG ATT GTC ATG GAC 3′ and reverse 5′ TCT GTC TGT CAC ATG GGT GAT GAA 3′ primers (Invitrogen, Carlsbad, CA, USA). For the eNOS gene we used forward 5′ CGG CAT CAC CAG GAA GAA GA 3′ and reverse 5′ GCC ATC ACC GTG CCC AT 3′ primers (Invitrogen). Real-time quantitative PCR was performed on a MIC qPCR Cycler (Bio Molecular Systems; Upper Coomera, Australia) using the Maxima SYBR Green/ROX qPCR master mix (Thermo Scientific, Cambridge, UK). B-actin was used as an internal control for the normalization of the examined angiogenic factors.

### 4.5. Immunocytochemistry/Immunohistochemistry

For cytoplasmatic staining, MNC were collected onto microscope glass slides by cytospins (2 × 10^4^ cells/each) and fixed by acetone at room temperature (RT). Bone marrow biopsy specimens were fixed in 10% neutral formalin solution for 24–36 h, then decalcified in EDTA buffer for 3 h and embedded in paraffin. The tissue sections were cut at 5 mm, heated at 56 °C for 60 min, then deparaffinized and rehydrated through a series of xylenes and alcohols followed by an epitope retrieval step. Samples were treated with 3% H_2_O_2_ solution in PBS to block endogenous peroxidase activity. The next step was incubation with an anti-IL6 antibody (Novocastra, Buffalo Grove, IL, USA) in a humidity chamber overnight at RT. Immunostaining was performed using the streptavidin-biotin technique (LSAB/HRP Kit, DAKO). Immunoreactivity was visualized with DAKO Liquid DAB^+^ Substrate/Chromogen System counterstained with Mayer’s hematoxylin (Merck, Whitehouse Station, NJ). For the negative control samples, normal serum and tris buffered saline (TBS) buffer (1:500) were pipetted without primary antibodies. Immunoreactive cells were analyzed and scored at five powered fields in each sample using a computer-supported imaging system (Analysis Pro 3.1) connected to a light microscope (Olympus AX70, Hamburg, Germany) with an objective magnification of 40.

### 4.6. Statistical Analysis

The one-way ANOVA and Dunnett post-test were applied using Prism 6 software (GraphPad Software Inc., San Diego, CA, USA). The results are expressed as the mean ± SEM, and differences at *p* < 0.05 were accepted as the level of significance.

## 5. Conclusions

The VEGF/VEGFR pathways are the most relevant regulators of angiogenesis and vasculogenesis and can also stimulate the proliferation, migration, and survival of tumor cells. Increased levels of pro-inflammatory cytokines are associated with poorer prognosis and shorter survival in patients with MPN, and therapy focused on the inflammatory cytokine profile may improve the quality of life and life expectancy of patients with MPN. We demonstrated the interaction between IL-6 and VEGF mediated by inflammation-related signaling pathways in MPN. VEGF promoted IL-6 productivity and enhanced the expression of related angiogenic factors that were conversely regulated by PI3K and mTOR signaling in MPN. VEGF can be a marker of MPN progression, while inflammation stimulated angiogenesis can be predisposed to fibrosis. The opposite regulation of the linked angiogenic factors by VEGF in PV and PMF can influence neovascularization and promote fibrosis in PMF.

## Data Availability

Not applicable.
